# Efficiency of structural brain networks mediates age-associated differences in executive functioning in older adults

**DOI:** 10.3389/fnagi.2025.1593868

**Published:** 2025-07-08

**Authors:** Geraldine Rodríguez-Nieto, Caroline Seer, Hamed Zivari Adab, Antonio Jimenez-Marin, Sima Chalavi, Amirhossein Rasooli, Jesús M. Cortés, Stefan Sunaert, Stephan P. Swinnen

**Affiliations:** ^1^Movement Control and Neuroplasticity Research Group, Group Biomedical Sciences, KU Leuven, Leuven, Belgium; ^2^KU Leuven Brain Institute (LBI), KU Leuven, Leuven, Belgium; ^3^Computational Neuroimaging Laboratory, BioBizkaia Health Research Institute, Barakaldo, Spain; ^4^Ikerbasque - Basque Foundation for Science, Bilbao, Spain; ^5^Department of Cell Biology and Histology, University of the Basque Country (UPV/EHU), Leioa, Spain; ^6^Department of Imaging and Pathology, KU Leuven, Leuven, Belgium

**Keywords:** aging, executive functions, structural connectivity, brain networks, graph theory, DWI, efficiency, segregation

## Abstract

**Introduction:**

Older age is associated with alterations in executive functioning (EF). Age-related alterations in the integrity of structural brain networks may contribute to EF decline, with potential consequences for independent living. Graph theory provides powerful metrics to examine the brain's structural connectome, but few studies have investigated the relationship of EF and structural brain networks, as described by graph-theoretical measures, in older adults. We aimed to investigate the mediatory role of network characteristics for the relationship between age and EF in older adults.

**Methods:**

Eighty-four older adults completed a battery of EF tasks to allow for the extraction of a latent Common-EF factor. White-matter tractograms were generated from diffusion neuroimaging using anatomically-constrained tractography (ACT) and spherical-deconvolution informed filtering of tractograms (SIFT2).

**Results:**

From the resulting networks, global efficiency (reflecting integration) as well as local efficiency (reflecting segregation) were calculated. Older age was associated with worse EF and decreased global and local efficiency. Both global and local efficiency were positively correlated with EF. Local efficiency mediated the negative correlation of age and EF, whereas no such relationship was found for global efficiency. Further regional efficiency analyses identified the nodes that contributed to the mediation effect of local efficiency.

**Discussion:**

These results shed light on the shared variability among the integrity of structural brain networks and EF at older age. A causal role of a reduced segregation in structural brain networks to support EF in older adults remains to be determined but would bear promising potential for preserving EF during aging.

## 1 Introduction

Executive functions (EFs) are higher-level mental processes that are believed to control lower-level operations, allowing for successful goal-directed behavior (Diamond, 2013; Friedman and Miyake, 2017). Age-related declines in EF (Ferguson et al., 2021; Fisk and Sharp, 2004; Rhodes, 2004) may have adverse consequences for wellbeing and functional independence. Several factors may contribute to age-related EF decline, including alterations in the brain white matter (Madden et al., 2009, 2012; Westlye et al., 2010). Specifically, interindividual differences in EF have been linked to decreases in white matter connectivity as seen in healthy aging (Coxon et al., 2012; Fjell et al., 2017; Gustavson et al., 2023; Hoagey et al., 2021; Li et al., 2020; Serbruyns et al., 2016; Tang et al., 2023; Ystad et al., 2011). White matter tracts across the brain have been linked to EF performance (Ribeiro et al., 2023). Accordingly, white matter microstructural alterations in regions supporting EF (i.e., a structural EF network) have been proposed as a mechanism underlying EF decline in aging (Bennett and Madden, 2014; Coxon et al., 2016; Fjell et al., 2017; Hoagey et al., 2021; Shen et al., 2020; Webb et al., 2020; Zahr et al., 2009).

Structural brain connectivity can be investigated using graph theoretical analysis (Bullmore and Sporns, 2009). Graph theoretical analysis describes brain networks as nodes and edges (i.e., pathways between nodes), and derives specific metrics that reflect different facets of the brain's network topology (Rubinov and Sporns, 2010; Sporns, 2013; Yeh et al., 2021). Within graph theoretical analysis, “efficiency parameters” describe the efficiency of information exchange within and between networks (Latora and Marchiori, 2001). Global efficiency (E_glob_) indicates the efficiency of parallel information transfer between all pairs of nodes in a network, and thus its integration (Cohen and D'Esposito, 2016). Regional efficiency (E_reg_) indicates, for every node in a network, how efficiently information can be transferred among its neighboring nodes when that node is removed. It thus reflects how much information transfer in a small area surrounding the node (i.e., a local subnetwork) is dependent on it (i.e., the efficiency of information transfer within this sub-network). Finally, local efficiency (E_loc_) denotes the average of E_reg_ across all nodes (Latora and Marchiori, 2001). Hence, E_glob_ is a measure of network integration, whereas E_reg_ and E_loc_ are measures of network segregation.

Graph theoretical analysis has revealed age-associated alterations in structural brain networks (Damoiseaux, 2017). Specifically, cross-sectional evidence links age to decreased global efficiency (Bi et al., 2021; Hinault et al., 2021; Li et al., 2020; Wen et al., 2011; Zhao et al., 2015; but see Gong et al., 2009) as well as regional and local efficiency (Bi et al., 2021; Gong et al., 2009; Li et al., 2020; Wen et al., 2011; Zhao et al., 2015). Overall, the literature suggests that structural brain networks deteriorate with increasing age, rendering them less efficient. This is consistent with an age-related “disconnection” of structural brain networks that may underlie age-associated cognitive decline (Bennett and Madden, 2014; Fjell et al., 2017; Madden et al., 2012; O'Sullivan et al., 2001).

Cognitive performance has been shown to correlate with the integrity of structural brain networks in older adults. For instance, Wen et al. (2011) found global network efficiency to be associated with processing speed, visuospatial abilities, and EF in older adults. In addition, Li et al. (2020) reported correlations with global and local network efficiency for both attention and EF in a similar population.

Taken together, the literature suggests age-related alterations in white matter networks, with potential consequences for EF. The present study was designed to investigate age-associated differences in global, local and regional structural networks efficiency and their contribution to age-associated performance differences in EF in healthy older adults, as indexed by a latent Common EF measure. The advantage of a latent EF metric is that it integrates several domains of EF and is not limited to one particular aspect of EF. In addition, it is more reliable and generalizable as compared to measures based on averaged z-scores obtained from single tasks per domain, as it reduces variability that is not specific to EF (Miyake et al., 2000; Miyake and Friedman, 2012). We hypothesized that (1) graph theoretical measures reflecting global and local efficiency of structural networks (see Methods) would be negatively related to age and positively to EF in older adults, and (2) the efficiency of information processing in the structural connectome (indexed by these graph theoretical measures) would account for age-associated differences in EF in older adults, as studied via mediation analyses. In addition, we aimed to identify the specific nodes from which connectivity show the strongest mediating association with the relationship among age, structural network efficiency and executive functioning.

## 2 Materials and methods

### 2.1 Participants and procedure

**Figure 1:**
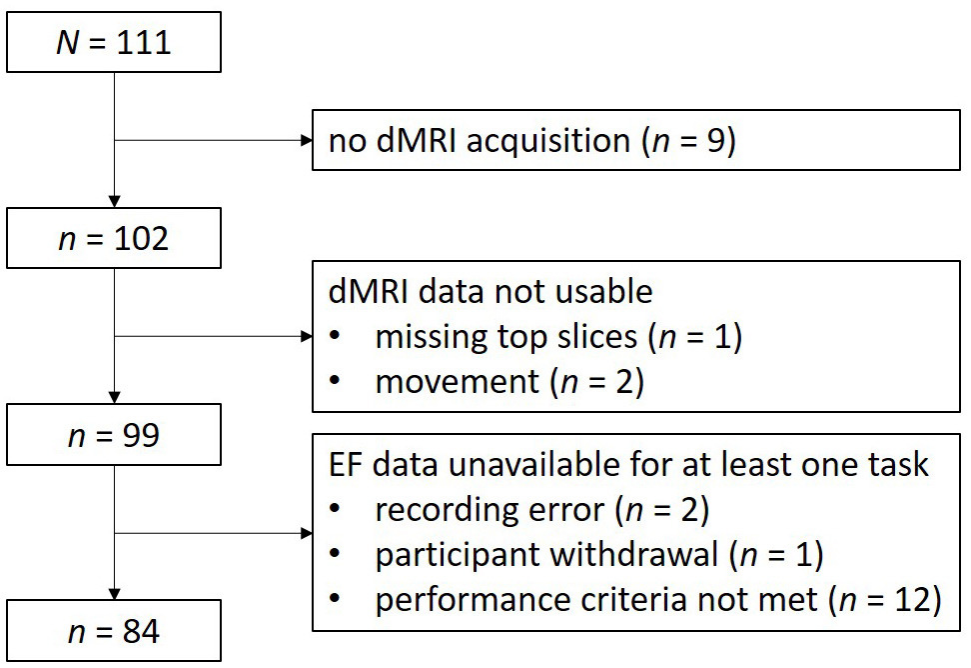
Flowchart depicting reasons for exclusion from analysis.

As part of a larger multimodal project investigating neural correlates of executive function (Seer et al., 2021, 2022), 111 older adults (aged 60 years and above) were recruited from the Leuven area (Belgium). All participants had normal or corrected-to-normal vision and reported no current intake of psychoactive medication, a current diagnosis of psychiatric/neurological disorder, and/or MRI contraindications. The present analyses included 84 participants (52 female, 32 male; 70 right-handed, 4 left-handed, 10 ambidextrous), between 60 and 85 years of age (*M* = 68.06, *SD* = 4.74), who had both high quality dMRI and EF data. Eligibility based on the performance on EF tasks has been described in detail elsewhere (Seer et al., 2021). In brief, participants showing signs of insufficient adherence to the task instructions (i.e., performance levels that did not differ from chance level on at least one EF task, *n* = 12 participants) were excluded. None of the participants in the final sample (*n* = 84; Figure 1) showed signs of mild cognitive impairment, as based on the Montreal Cognitive Assessment (MoCA; *M* = 27.71, *SD* = 1.82, range: 24-30 (cutoff = 23/30, Carson et al., 2018); (Nasreddine et al., 2005). Subjective cognitive complaints were not assessed. The average number of education years was 18.06 (*SD* = 2.66; range: 11-24) and the average level of crystallized intelligence on the Peabody Picture Vocabulary Test (PPVT) was 109.50 (*SD* = 8.76, range: 82-125; Horn and Cattell, 1967; Schlichting, 2005). The study was reviewed and approved by the Ethics Committee Research UZ/KU Leuven (study number 61,577). All participants provided written informed consent to participate and were offered a compensation of € 100. The dataset is openly available on https://osf.io/hxr38/files/osfstorage.

The study protocol is described in detail elsewhere (Seer et al., 2021). Participants completed three sessions: (1) a first behavioral session, where they completed background assessments and questionnaires as well as three computerized EF tasks, (2) a second behavioral session, where the remaining six computerized EF tasks were completed, and (3) a neuroimaging session. Participants completed the whole experiment on average within ~2 weeks.

### 2.2 Executive functioning tasks

Participants completed a comprehensive computerized battery in OpenSesame version 3.2.6 (Mathôt et al., 2012) of nine neuropsychological tasks across two test days, following a protocol similar to Friedman et al. (2016). This test battery was designed to cover three key domains of EF, i.e., inhibition (suppressing unwanted actions), shifting (switching between mental operations), and updating (managing working memory content). Every domain was tapped by three tasks. The inhibition domain was tapped by antisaccade, number-Stroop, and stop-signal tasks. In the antisaccade task, participants are presented with salient visual cues and need to avoid automatic saccades toward that stimulus. In the number-Stroop task, participants need to avoid reading out a number from a string of numbers and instead report how many numbers the string contained. In the stop-signal task, participants should withhold a prepotent motor response to a simple categorization task when cued to do so. The shifting domain was tapped by category switch, color-shape, and number-letter tasks. In all of these, participants are asked to switch back and forth between two tasks according to a visual task cue. In the category switch task, participants are cued to categorize words as either denoting an animate vs. an inanimate object (animacy task) or as describing an object that is larger vs. smaller than a football (size task). In the color-shape task, participants are asked to categorize stimuli either according to their shape (triangle vs. circle; shape task) or according to their color (red vs. green; color task). In the number-letter task, participants are presented with pairs of letters and numbers and are cued to categorize these pairs either regarding the letter being a vowel vs. a consonant (letter task) or regarding the number being odd vs. even (number task). The updating domain was tapped by digit span, keep track, and spatial 2-back tasks. In the digit-span task, participants are asked to repeat strings of numbers either in forward or in backward order, with the length of the strings increasing until the participant fails to respond correctly. In the keep track task, participants are asked to attend to a stream of words from different categories (e.g., countries, colors) and recall the last word of each category, with varying numbers of categories to keep track of. In the spatial 2-back task, participants are asked to watch a sequence of dots flashing on different locations scattered across the computer screen and to indicate, for every dot, whether the dot in that same location has been highlighted two trials before the current one. The task order was fixed to minimize between-subject variability (e.g., due to learning or fatigue effects) and hence facilitate latent variable extraction (day 1: stop-signal, category switch, digit span; day 2: color-shape, keep track, anti-saccade, spatial 2-back, number-Stroop, number-letter; Friedman et al., 2016). The rationale for this particular task order, individual task parameters, and calculation of performance scores were described in detail elsewhere (Seer et al., 2021).

A single measure of EF was derived from the common and specific EF variance, using the unity/diversity framework (Friedman and Miyake, 2017; Miyake et al., 2000; Miyake and Friedman, 2012). The nine performance scores for the tasks described above were entered into a confirmatory factor analysis in lavaan 0.6–7 (Rosseel, 2012), where a “Common EF” factor represented the shared variance by all tasks while “shifting-specific” and “updating-specific” factors represented the residual variability from shifting and updating tasks (Miyake and Friedman, 2012; Seer et al., 2021). Note that after accounting for Common EF variability, there is usually no residual variability left to be captured by an “inhibition-specific” factor; this was also the case in the current dataset (see also Friedman and Miyake, 2017; Seer et al., 2021). In the context of the present study, the “Common EF” factor score was used as the main variable of interest when assessing the interrelations between EF, Age, and brain/graph metrics in this cohort of older adults. Factor loadings and model fit indices are provided in the supplement (Supplementary Table S1). Note that this procedure also yielded shifting-specific and updating-specific EF factors. Although these factors were not of interest for the present study, we executed exploratory analyses for completeness.

### 2.3 MRI acquisition

MRI data were acquired on a Philips Achieva 3.0T MRI system equipped with a 32-channel head coil. A high-resolution three-dimensional T1-weighted (T1W) structural image was collected, using a magnetization-prepared rapid gradient echo (MPRAGE) sequence with the following parameters: TR/TE = 5.6/2.5 ms; flip angle = 8°; voxel size = 0.9 × 0.9 × 0.9 mm^3^; field of view = 256 × 240 × 187.2 mm^3^; 208 sagittal slices; sensitivity encoding (SENSE) = 2; total scan time = ~ 6 min. Diffusion MRI data were acquired using a single-shot echo planar imaging sequence with the following parameters: dMRI volumes with b-values = 700 s/mm^2^ (16 gradient directions), 1,200 s/mm^2^ (30 gradient directions), and 2,800 s/mm^2^ (50 gradient directions); 6 interspersed volumes without diffusion weighting (b = 0 s/mm^2^); flip angle = 90°; phase-encoding direction = posterior to anterior (PA); field of view = 240 × 240 × 140 mm^3^; voxel size = 2.5 × 2.5 × 2.5 mm^3^, TE/TR = 74/5,000 ms; multiband factor = 2; SENSE = 2; matrix size = 96 × 96; 56 transverse slices; total scan time = ~ 9 min. We also acquired five b = 0 s/mm^2^ images with reversed phase encoding (AP) for the purpose of susceptibility-induced distortion correction.

### 2.4 MRI processing

**Figure 2:**
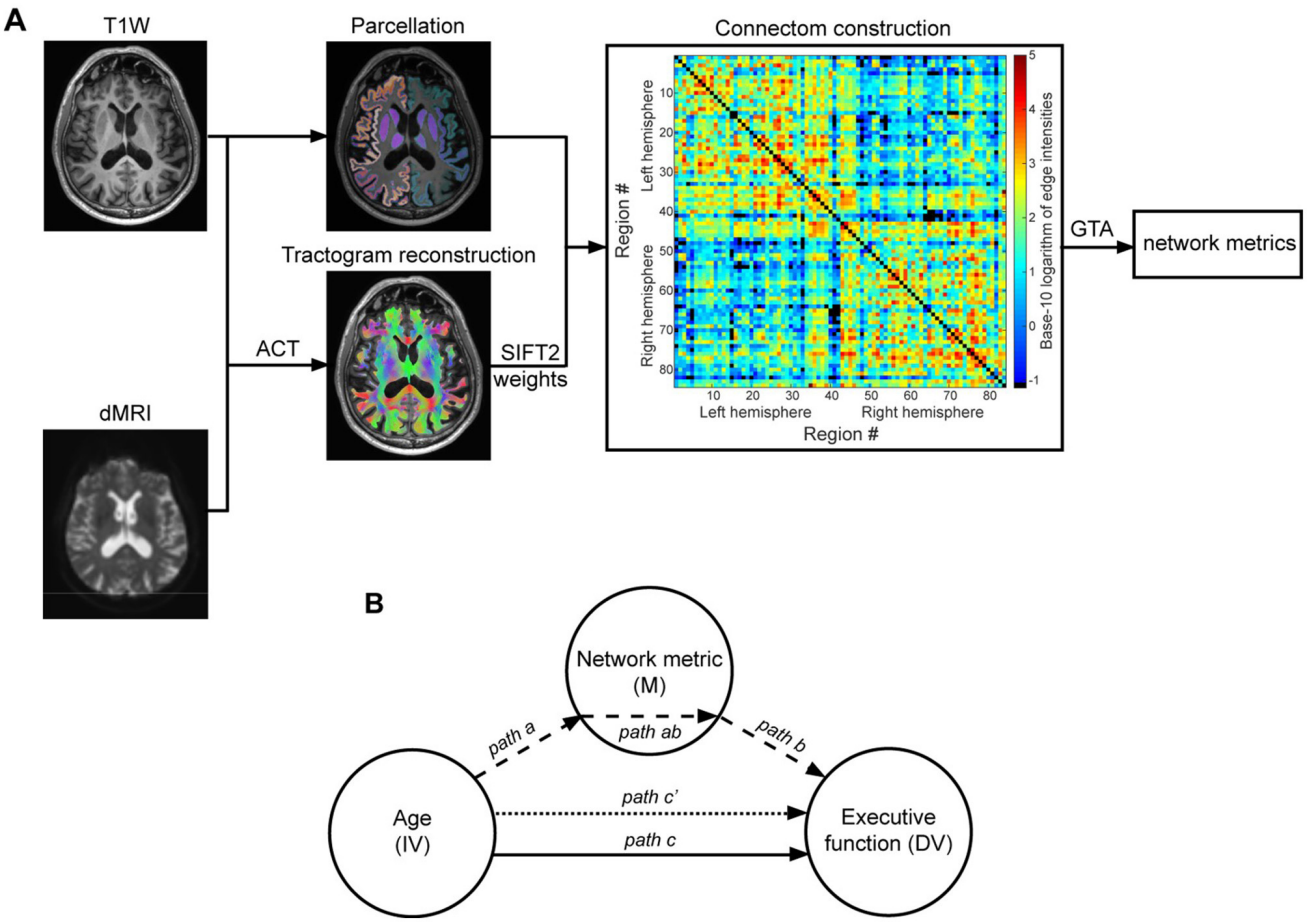
General overview of applying graph theoretical analysis to study brain networks **(A)** and overview of the mediation model **(B)**. **(A)** The anatomical constrained tractography (ACT) framework was applied to the preprocessed dMRI data and T1W image of an exemplary participant to reconstruct the whole brain tractogram (overlaid on T1W image). The streamlines' weights obtained via SIFT2 (spherical-deconvolution informed filtering of tractograms) were then used in conjunction with the brain nodes image, obtained by parcellating the T1W image, to construct an 84 × 84 weighted and symmetrical connectivity matrix. The color bar shows connection strength in logarithmic scale and missing/removed connections are in black. Graph theoretical analysis was used to calculate the weighted version of network topology metrics of interest (here: global efficiency, local efficiency, regional efficiency). **(B)** The Mediation model was used to determine whether the age-related differences in EF in older adults are mediated by alterations in network metrics. Path *c* (solid line) = total effect of age (IV = independent variable) on executive function (DV = dependent variable). This total effect was, per metric of interest, separated into two distinct pathways: (1) path *ab* (dashed arrows) = indirect (mediation) effect, with path *a* reflecting the effect of age on the network metric (M = mediator variable) and path *b* reflecting the effect of network metric on executive function; (2) path *c*′ (dotted arrow) = direct effect, i.e., the effect of age on executive function independent of its effect through the network metric.

The MRtrix3 (Tournier et al., 2019) standard structural connectome construction pipeline (Smith and Connelly, 2019) available at https://github.com/BIDS-Apps/MRtrix3_connectome and described in detail elsewhere (Civier et al., 2019; Smith et al., 2015b; Yeh et al., 2016, 2019), was applied to dMRI and T1W data (see Figure 2A for a general overview of the pipeline). Where necessary, this pipeline also incorporates commands from FSL (Jenkinson et al., 2012) and Freesurfer (Fischl, 2012) software packages. Brain parcellation was performed according to the Desikan atlas (Desikan et al., 2006), which is the default atlas used by Freesurfer.

In brief, dMRI data were denoised (Veraart et al., 2016), Gibbs unringed (Kellner et al., 2016), and corrected for eddy current distortions, motion, and susceptibility induced distortions (Andersson et al., 2003, 2016, 2017; Andersson and Sotiropoulos, 2016). Three-tissue response functions representing single-fiber white matter, gray matter and cerebrospinal fluid were obtained from the corrected dMRI data using an unsupervised approach (Dhollander et al., 2016). Three-tissue constrained spherical deconvolution (CSD) was performed for each participant, using the averaged (across all participants) response functions for each tissue type with the multi-shell multi-tissue CSD algorithm (Jeurissen et al., 2014), resulting in the white matter fiber orientation distribution (FOD) for each voxel. Joint bias field correction and global intensity normalization of the 3-tissue parameters was performed in the log-domain (Dhollander et al., 2021). Subject's T1W image was also registered to the mean b = 0 s/mm^2^ (corrected) image via rigid-body transformation (Bhushan et al., 2015).

Following the initial processing, tractograms were generated. Thus, for each participant, the 2^nd^-order integration over FODs algorithm (iFOD2; Tournier et al., 2010) and the anatomically-constrained tractography (ACT; Smith et al., 2012) with dynamic seeding (Smith et al., 2015a), FOD amplitude threshold 0.06, step size of 1.25 mm, length of 5–250 mm, and backtracking (Smith et al., 2012) were used to generate 10 million probabilistic streamlines. Furthermore, each streamline was assigned a weight, computed using the spherical-deconvolution informed filtering of tractograms (SIFT2; see Smith et al., 2015a). Based on each participant's tractogram, an individual connectome was computed using 84 regions-of-interest parcellated in native space [cortex and cerebellum: Dale et al. (1999); Desikan et al. (2006); subcortical regions: Patenaude et al. (2011); see Smith et al. (2015a)], with connection strengths calculated by summing the weights of the relevant streamlines scaled by the proportionality coefficient (Smith et al., 2015a). These 84 nodes were used for further analyses. To allow for a better understanding of the distribution of regional differences, the nodes were grouped into seven larger areas (frontal, parietal, temporal, occipital, insula-cingulate, subcortical (including hippocampus), and cerebellum for the analysis of regional efficiency (see below). This categorization followed the grouping of cortical regions suggested by Klein and Tourville (2012), which is based on the Desikan atlas (Desikan et al., 2006). Intra-regional connection strengths were set to zero (Rubinov and Sporns, 2010).

### 2.5 Global and local efficiency as putative measures of integration and segregation

The Brain Connectivity Toolbox (Rubinov and Sporns, 2010), implemented in MATLAB (The MathWorks Inc., Natick, MA), was used to compute weighted, undirected network metrics including, global efficiency (E_glob_: $E=\;\frac{1}{n}\underset{i\in N}{\sum }E_{i}=\;\frac{1}{n}\underset{i\in N}{\sum }\frac{\underset{j\in N,j\neq i}{\sum }d_{ij}^{-1}}{n-1}$, where *E*_*i*_ is the efficiency of node *i*; Rubinov and Sporns, 2010), regional efficiency (E_reg_), and local efficiency (E_loc_: $E_{loc}=\frac{1}{n}\underset{i\in N}{\sum }E_{loc,i}=\;\frac{1}{n}\underset{i\in N}{\sum }\frac{\underset{j,h\in N,j\neq i}{\sum }{a_{ij}a_{ih}\left[d_{jh}\left(N_{i}\right)\right]}^{-1}}{k_{i}\left(k_{i}-1\right)}$, where *E*_*loc, i*_ is the local efficiency of node *i*, and *d*_*jh*_(*N*_*i*_) is the length of the shortest path between *j* and *h*, that contains only neighbors of *i*; Rubinov and Sporns, 2010). Please note that E_loc_ is the average of E_reg_ across all nodes. In this study, the recommended version described in Wang et al. (2017) was used to calculate E_reg_ as it is a true generalization of the binary variant.

E_glob_ measures how efficient the parallel information transfer (flow) in the network is and thus is an index of network integration. E_reg_ and E_loc_ (i.e., the average of E_reg_ across all nodes) measure the efficiency of the communication amongst the first neighbors of a node when that node is removed. These metrics indicate how well a network tolerates faults and thus are indices of network segregation (Latora and Marchiori, 2001). In other words, the intercommunicability of any two nodes in the network is reflected in the network integration, or global efficiency. In contrast, the efficiency of specific clusters is reflected in the network segregation, or regional and local efficiency (see also Cohen and D'Esposito, 2016).

We decided to focus on efficiency metrics because these are reflective of the integration and segregation of the network's connectivity and because these metrics have been associated with age and/or to executive functioning in older adults (Li et al., 2020; Madden et al., 2020; Wen et al., 2011; Zhao et al., 2015). Furthermore, regional efficiency supports the identification of relevant nodes in the association among efficiency, age and executive functioning, rendering higher specificity. Nonetheless, we admit that other network properties may also be relevant. Accordingly, we included analogous supplementary analyses of other network metrics (density, clustering, modularity and strength).

### 2.6 Statistical analysis

Kolmogorov-Smirnov tests did not show significant deviations from normality, which was confirmed by visual inspection for the variables of interest. Partial (Pearson) correlation analyses controlling for sex and education were used to investigate the bivariate associations between age, EF, and network metrics. To examine whether age-associated variations in network metrics contribute to age-associated differences in EF in a cohort of older adults, mediation analyses were performed (MacKinnon et al., 2007). To this end, the commonly-used simple 3-path mediation model (Baron and Kenny, 1986), implemented in the PROCESS V4.0 plugin (Hayes and Rockwood, 2017) developed for IBM SPSS (V28.0 for Windows), was used (Figure 2B). Multiple comparisons were controlled using a False Discovery Rate (FDR) correction (Benjamini and Hochberg, 1995).

In this model, the total effect (path *c*) of independent variable (IV) on dependent variable (DV) was separated into two distinct pathways (see Figure 2): (1) indirect (mediation) effect (path *ab*) with path *a* reflecting the effect of IV on the mediator variable (M) and path *b* reflecting the effect of mediator variable on DV while controlling for IV; (2) direct effect of IV on DV (path *c'*), i.e., the effect of IV on DV independent of its effect through the mediator variable. Of particular interest was the indirect effect (path *ab*), since a significant indirect effect would indicate significant mediation by the mediator variable used in the model. This was accomplished using 5,000 bootstrap samples to determine bias-corrected confidence intervals for the indirect effects. Accordingly, indirect effects with 95% confidence intervals excluding zero were regarded as significantly mediating the relation between IV and DV. In this study, age, graph metrics, and common EF were, respectively, assigned as IV, M and DV. Sex and education were included as nuisance variables in all mediation models.

We conducted supplementary analyses for global metrics other than efficiency (density, clustering, modularity, and strength) to investigate whether these variables were related to age and common EF and to examine whether they mediated the age-common EF relationship. These analyses are presented in the Supplementary material (Supplementary Tables S3–S9).

## 3 Results

### 3.1 Age-associated differences in executive functioning in older adults

**Figure 3:**
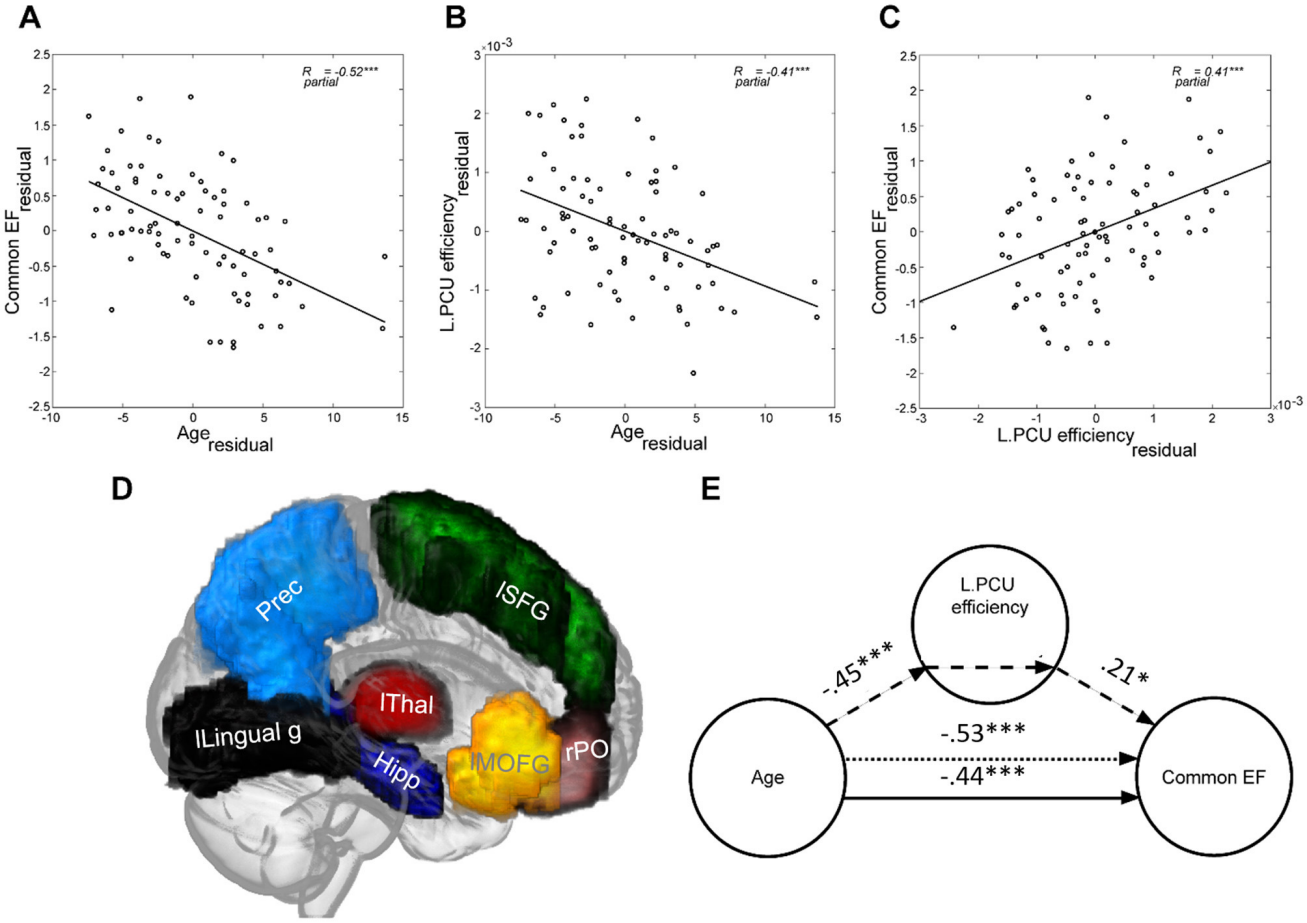
Age was negatively associated with both **(A)** Common EF and **(B)** Left Precuneus (as an exemplary region) efficiency in older adults. **(C)** Higher Left Precuneus efficiency was related to better Common EF performance. **(D)** The 3D representation of brain regions for which the efficiency significantly mediated the age-associated decline in Common EF in older adults (listed in **Table 3**) is shown on the glass brain. **(E)** The standardized path coefficients of the mediation model used for the same region as in **(B, C)** (i.e., Left Precuneus) are shown. In all plots sex and education are controlled. lSFG, left Superior Frontal Gyrus; lMOFG, left Medial Orbitofrontal Gyrus; Prec, bilateral Precuneus; lLingual g, left Lingual gyrus; Hipp, bilateral Hippocampus; lThal, left Thalamus; rPO, right Pars Orbitalis. ***p < 0.001; **p < 0.005; *p < 0.050; CI: 95% bias-corrected bootstrapped confidence interval.

Within this cohort of older adults, age was significantly correlated with Common EF (*r* = −0.52, *p* < 0.001; note that this correlation remained significant when sex and education were omitted as controlling variables, *r* = −0.50, *p* < 0.001). The negative correlation coefficient indicates older age to be associated with lower Common EF scores (i.e., poorer EF; Figure 3A).

### 3.2 Age-associated differences in brain efficiency in older adults

Investigations into brain efficiency in this cohort of older adults showed significant negative correlations for global (E_glob_: 0.026 ± 0.004; *r* = −0.43, *p* < 0.001) and local (E_loc_: _0.0_03 ± 0.0005; *r* = −0.43, *p* < 0.001) efficiency parameters with age. Omitting sex and education as controlling variables did not qualitatively change these results (E_glob_: *r* = −0.42, *p* < 0.001; E_loc_: *r* = −0.41, *p* < 0.001). The negative correlation coefficients indicated a decrease in global (i.e., efficiency of information transfer for the entire brain) and local (i.e., average efficiency of information transfer in local subnetworks) efficiency of the brain with increasing age. Furthermore, E_reg_ of 51% (43 out of 84) of the brain regions showed a significant (FDR corrected) negative correlation with age, indicating a decrease in regional efficiency as age increases (Table 1, Figure 3B for an example). To obtain a better understanding of how these regions are distributed, the individual nodes were assigned to one of seven areas including frontal (22 nodes), parietal (10 nodes), temporal (18 nodes), occipital (8 nodes), insula-cingulate (10 nodes), subcortical (including hippocampus; 14 nodes), and cerebellum (2 nodes), according to a predefined categorization (Klein and Tourville, 2012). This assignment revealed that 18 of the negatively correlated regions were located in frontal areas, 9 in parietal areas, 9 in temporal areas, 3 in occipital areas, and 4 in below-/sub-cortical areas. No significant region was found in insula-cingulate and cerebellum. No significant positive correlation with age was found for E_reg_ of any brain region. Finally, supplementary analyses showed that density, clustering and strength (but not modularity) were negatively related to age (Supplementary Table S4).

**Table 1:** Regions with significant age-E_reg_ association (controlled for sex and years of education) are listed according to hemisphere, lobe, and ascending order of *p*-value (FDR critical *p* = 0.026).

**Region**	**Lobe/Area**	***r***	***p***
**Left hemisphere**			
Pars orbitalis	Frontal	−0.44	< 0.001
Pars opercularis	Frontal	−0.43	< 0.001
Rostral middle frontal gyrus	Frontal	−0.42	< 0.001
Pars triangularis	Frontal	−0.39	< 0.001
Superior frontal gyrus	Frontal	−0.33	0.002
Lateral orbitofrontal gyrus	Frontal	−0.33	0.002
Precentral gyrus	Frontal	−0.31	0.004
Caudal middle frontal	Frontal	−0.27	0.014
Medial orbitofrontal gyrus	Frontal	−0.26	0.017
Superior parietal gyrus	Parietal	−0.47	< 0.001
Precuneus	Parietal	−0.41	< 0.001
Supramarginal gyrus	Parietal	−0.38	< 0.001
Inferior parietal gyrus	Parietal	−0.37	0.001
Postcentral gyrus	Parietal	−0.33	0.002
Middle temporal gyrus	Temporal	−0.34	0.002
Superior temporal gyrus	Temporal	−0.31	0.006
Transverse temporal gyrus	Temporal	−0.27	0.014
Inferior temporal gyrus	Temporal	−0.25	0.025
Lateral occipital gyrus	Occipital	−0.31	0.004
Hippocampus	Subcortical	−0.38	< 0.001
Thalamus	Subcortical	−0.34	0.002
**Right hemisphere**			
Rostral middle frontal gyrus	Frontal	−0.54	< 0.001
Pars triangularis	Frontal	−0.44	< 0.001
Pars orbitalis	Frontal	−0.43	< 0.001
Superior frontal gyrus	Frontal	−0.42	< 0.001
Precentral gyrus	Frontal	−0.34	0.002
Caudal middle frontal gyrus	Frontal	−0.32	0.004
Pars opercularis	Frontal	−0.32	0.004
Lateral orbitofrontal gyrus	Frontal	−0.26	0.021
Paracentral gyrus	Frontal	−0.25	0.021
Superior parietal gyrus	Parietal	−0.47	< 0.001
Precuneus	Parietal	−0.46	< 0.001
Postcentral gyrus	Parietal	−0.43	< 0.001
Inferior parietal gyrus	Parietal	−0.38	< 0.001
Entorhinal cortex	Temporal	−0.37	< 0.001
Superior temporal gyrus	Temporal	−0.37	< 0.001
Inferior temporal gyrus	Temporal	−0.35	0.001
Fusiform gyrus	Temporal	−0.26	0.016
Banks of the superior temporal sulcus	Temporal	−0.25	0.025
Lateral occipital gyrus	Occipital	−0.37	< 0.001
Cuneus	Occipital	−0.27	0.013
Hippocampus	Subcortical	−0.47	< 0.001
Accumbens	Subcortical	−0.41	< 0.001

*r*-values are rounded to two decimals. Assignment of lobes/areas according to Desikan et al. (2006).

### 3.3 Relationship between brain efficiency and executive functioning in older adults

The E_glob_ (*r* = 0.33, *p* = 0.001) and E_loc_ (*r* = 0.37, *p* = 0.001) were significantly correlated with Common EF in older adults. Omitting sex and education as control variables did not qualitatively change these results (E_glob_: *r* = 0.35, *p* = 0.001, E_loc_: *r* = 0.39, *p* < 0.001). The positive correlation coefficients indicated that superior Common EF was associated with higher global (i.e., efficiency of information transfer for the entire brain) and local (i.e., average efficiency of information transfer in local subnetworks) efficiency of the brain. Moreover, E_reg_ of 55% (46 out of 84) of the brain regions showed a significant (FDR corrected) positive correlation with Common EF factor (Table 2, Figure 3C for an example). Thus, higher regional efficiency corresponded to better Common EF. Assigning brain regions to the different areas (Klein and Tourville, 2012) revealed that 16 of the positively correlated regions were located in frontal areas, 10 in parietal areas, 7 in temporal areas, 4 in occipital areas, and 9 in below-/sub-cortical areas. No significant region was found in insula-cingulate and cerebellum.

**Table 2:** Regions with significant Common EF-E_reg_ association (controlled for sex and years of education) are listed according to hemisphere, lobe, and ascending order of *p*-value (FDR critical *p* = 0.027).

**Region**	**Lobe/Area**	***r***	***p***
**Left hemisphere**			
Superior frontal gyrus	Frontal	0.35	0.001
Medial orbitofrontal gyrus	Frontal	0.35	0.001
Rostral middle frontal gyrus	Frontal	0.34	0.002
Lateral orbitofrontal gyrus	Frontal	0.33	0.003
Pars opercularis	Frontal	0.31	0.005
Pars orbitalis	Frontal	0.29	0.007
Frontal pole	Frontal	0.26	0.017
Precentral gyrus	Frontal	0.25	0.022
Precuneus	Parietal	0.41	< 0.001
Superior parietal gyrus	Parietal	0.33	0.002
Inferior parietal gyrus	Parietal	0.31	0.004
Supramarginal gyrus	Parietal	0.29	0.007
Postcentral gyrus	Parietal	0.27	0.013
Middle temporal gyrus	Temporal	0.31	0.004
Transverse temporal gyrus	Temporal	0.28	0.011
Banks of the Superior Temporal Sulcus	Temporal	0.25	0.024
Superior temporal gyrus	Temporal	0.25	0.025
Lingual gyrus	Occipital	0.38	< 0.001
Cuneus	Occipital	0.26	0.018
Hippocampus	Subcortical	0.37	< 0.001
Thalamus	Subcortical	0.36	< 0.001
Putamen	Subcortical	0.29	0.007
Accumbens	Subcortical	0.28	0.012
Caudate	Subcortical	0.26	0.019
**Right hemisphere**			
Pars orbitalis	Frontal	0.36	< 0.001
Superior frontal gyrus	Frontal	0.33	0.003
Pars triangularis	Frontal	0.31	0.005
Medial orbitofrontal gyrus	Frontal	0.31	0.006
Rostral middle frontal gyrus	Frontal	0.29	0.008
Lateral orbitofrontal gyrus	Frontal	0.28	0.011
Frontal pole	Frontal	0.28	0.012
Precentral gyrus	Frontal	0.27	0.012
Precuneus	Parietal	0.42	< 0.001
Superior parietal gyrus	Parietal	0.29	0.008
Postcentral gyrus	Parietal	0.27	0.013
Posterior cingulate	Parietal	−0.25	0.025
Inferior parietal gyrus	Parietal	0.25	0.027
Inferior temporal gyrus	Temporal	0.33	0.002
Fusiform gyrus	Temporal	0.29	0.008
Middle temporal gyrus	Temporal	0.28	0.011
Cuneus	Occipital	0.29	0.007
Lateral occipital gyrus	Occipital	0.27	0.016
Hippocampus	Subcortical	0.41	< 0.001
Thalamus	Subcortical	0.32	0.003
Accumbens	Subcortical	0.31	0.005
Caudate	Subcortical	0.25	0.023

*r*-values are rounded to two decimals. Assignment of lobes/areas according to Desikan et al. (2006).

Supplementary analyses showed that neither the shifting-specific nor the updating-specific factor were correlated with the efficiency metrics (Supplementary Table S10). In addition, density, clustering and strength (but not modularity) were positively related to Common EF (Supplementary Table S4).

### 3.4 Mediation of age-associated differences in brain efficiency on age-associated differences in executive functioning in older adults

Using E_glob_ as a mediator, no significant mediation effect on age-associated decrease in Common EF (β_*c*_ = −0.53, *p* < 0.001) was found (β_*ab*_ = −0.06, CI: [-0.17, 0.02]; β_*a*_ = −0.44, *p* < 0.001; β_*b*_ = 0.13, *p* = 0.21, $\beta_{c^{\prime }}$ = −0.47, *p* < 0.001; sex standardized coefficient = −0.19, *p* = 0.05; education standardized coefficient = 0.09, *p* = 0.37).

E_loc_ significantly mediated the age-associated differences in Common EF in this cohort of older adults (β_*c*_ = −0.53, *p* < 0.001; β_*ab*_ = −0.07, CI: [−0.16,−0.002]; β_*a*_ = −0.44, *p* < 0.001; β_*b*_ = 0.18, *p* = 0.09; $\beta_{c^{\prime }}$ = −0.45, *p* < 0.001; sex standardized coefficient = −0.19, *p* = 0.05; education standardized coefficient = 0.09, *p* = 0.37).

To identify for which brain regions E_reg_ mediated the age-associated differences in EF, we restricted the mediation analysis to those 38 regions showing significant associations with both age and Common EF in previous analyses (i.e., common regions in Tables 1, 2). The result of this analysis indicated that the age-associated differences in regional efficiency of bilateral precuneus (parietal), bilateral hippocampus (below/sub-cortical), left superior frontal gyrus (frontal), left medial orbitofrontal gyrus (frontal), left thalamus (below/sub-cortical), left lingual gyrus (occipital), and right pars orbitalis (frontal) significantly contributed to the differences in Common EF in older adults (Table 3, Figures 3D, E for an example).

**Table 3:** Brain regions with E_reg_ significantly mediating the age-associated differences in Common EF in older adults.

**Region**	**Lobe/Area**	**β_c_**	** $\beta_{{\text{c}}^{\text{′}}}$ **	**β_ab_**	**Boot SE**	**Boot LLCI**	**Boot ULCI**	**β_a_**	**β_b_**
**Left hemisphere**									
Superior frontal gyrus	Frontal	−0.53^***^	−0.46^***^	−0.07	0.04	−0.15	−0.01	−0.34^**^	0.21^*^
Medial orbitofrontal gyrus	Frontal	−0.53^***^	−0.47^***^	−0.06	0.04	−0.14	−0.01	−0.27^*^	0.23^*^
Precuneus	Parietal	−0.53^***^	−0.44^***^	−0.09	0.04	−0.19	−0.02	−0.45^***^	0.21^*^
Lingual gyrus	Occipital	−0.53^***^	−0.47^***^	−0.07	0.04	−0.14	−0.002	−0.25^*^	0.26^**^
Hippocampus	Subcortical	−0.53^***^	−0.46^***^	−0.07	0.04	−0.16	−0.001	−0.39^***^	0.18 ^†^
Thalamus	Subcortical	−0.53^***^	−0.46^***^	−0.07	0.04	−0.16	−0.001	−0.34^***^	0.21^***^
**Right hemisphere**									
Pars orbitalis	Frontal	−0.53^***^	−0.46^***^	−0.07	0.04	−0.16	−0.002	−0.45^***^	0.17^†^
Precuneus	Parietal	−0.53^***^	−0.42^***^	−0.11	0.05	−0.22	−0.01	−0.48^***^	0.23^*^
Hippocampus	Subcortical	−0.53^***^	−0.43^***^	−0.09	0.05	−0.21	−0.003	−0.48^***^	0.21^†^

The regions are listed according to hemisphere and lobe. Zero outside the CI indicates significance of the mediation effect (β_ab_). We note that β_c_ (i.e., total effect of age on Common EF) is the same in all models.

β, standardized regression coefficient; CI, bias-corrected 95% confidence interval; Boot SE, LLCI, and ULCI, mediation effect's standard error, lower, and upper limit of CI obtained by bootstrapping (n = 5,000). Assignment of lobes/areas according to Desikan et al. (2006).

^***^ p < 0.001; ^**^ p < 0.005; ^*^ p < 0.050; ^†^ p < 0.100.

Supplementary analyses showed that from the global metrics commonly related to age and Common EF (density, clustering and strength), only clustering was a significant mediator of the relationship between age and Common EF (Supplementary Table S5).

## 4 Discussion

This study addressed age-associated differences in global, local and regional efficiency of structural connectivity and their contribution to age-associated differences in EF in healthy older adults. We performed latent variable modeling for the assessment of EF along with the most recent state-of-the-art techniques for structural connectome construction. We found negative associations between age and global (i.e., efficiency of information transfer for the entire brain), local (i.e., average efficiency of information transfer in local subnetworks), and—for a range of brain areas—regional network efficiency metrics of structural brain networks in older adults. In addition, better EF performance was associated with higher global, local, and—for a range of brain areas—regional network efficiency. Importantly, we found that local efficiency and regional efficiency of particular nodes mediated age-associated interindividual variations in EF in older adults. In contrast, global efficiency was not a significant mediator, which may suggest that the lower performance in EF with age in older adults is mediated by a decreased segregation rather than a decreased integration.

### 4.1 Age-associated differences in executive functioning in older adults

Our results revealed that age was negatively related to EF—indicated by a latent factor reflecting general executive abilities—in a group of older adults. In other words, relatively younger age within the group of older adults studied here (aged between 60 and 85 years) was associated with better EF performance, which is in line with previous research (Ferguson et al., 2021; Maldonado et al., 2020). Note that this finding does not demonstrate a decline in EF that is attributable to the aging process itself, but it does reflect poorer EF performance in older in comparison to relatively younger individuals (in the population of older adults).

### 4.2 Age-associated differences in brain efficiency in older adults

Normal brain function implies two co-existing fundamental aspects of functional organization, namely segregation and integration of information in brain networks. Segregation refers to the ability for specialized processing within densely interconnected groups of the brain and integration refers to the ability to combine the specialized information from distributed brain networks (Rubinov and Sporns, 2010).

In this study, we focused on graph theory metrics of global and local efficiency of structural brain networks as measures of integrated and segregated information transfer, respectively. Note that graph theory also offers alternative metrics to cover network segregation and integration characteristics, such as clustering and transitivity (segregation) and characteristic path length (integration; Rubinov and Sporns, 2010; Farahani et al., 2019). The latter is highly related to the global efficiency measure (Madole et al., 2023), as both metrics use the estimation of the shortest path among nodes.

Age showed significant negative associations with both ‘'global” (i.e., efficiency of information transfer for the entire brain) and ‘'local” (i.e., average efficiency of information transfer in local subnetworks) efficiency. In other words, the global and local efficiency of structural brain networks was lower as a function of age (note that this does not imply an effect of *aging*, i.e., change over time). These findings are in line with earlier studies, demonstrating lower efficiency of structural brain networks at higher age (Bi et al., 2021; Gong et al., 2009; Hinault et al., 2021; Li et al., 2020; Wen et al., 2011; Zhao et al., 2015).

Both global efficiency and local efficiency both are metrics of information transfer within the brain, but they reflect different aspects: global efficiency reflects how well a network is integrated, whereas local efficiency reflects how clearly subnetworks are segregated (Cohen and D'Esposito, 2016; Latora and Marchiori, 2001; Rubinov and Sporns, 2010). Both global and local efficiency are decreased in older age. To the extent that network integration and network segregation are important for optimal functioning, our results may be suggestive of age-associated differences in successful information processing. Such a disruption of structural networks has been interpreted as a network “disconnection,” possibly underlying age-associated EF differences (see above; Bennett and Madden, 2014; Fjell et al., 2017; Madden et al., 2012; O'Sullivan et al., 2001).

Several studies have suggested an association of older age and decreased segregation of functional networks (i.e., increased connectivity *between* functional networks; Antonenko and Flöel, 2014; Damoiseaux, 2017; Deery et al., 2023; King et al., 2018). Our current results complement those findings in that they reveal lower efficiency of information transfer in structural brain networks in older individuals.

In this cohort of older adults, we found lower efficiency to be associated with older age in an aggregated measure across all 84 brain regions under investigation. In addition, more fine-grained analyses revealed that these effects were found across the whole brain territory, except for limbic (insula-cingulate) regions and the cerebellum. The most prominent effects were observed in frontal, parietal, and subcortical (including hippocampus) regions. The negative association between age differences and network efficiency for fronto-parietal and subcortical (including hippocampus) regions in the present dataset is roughly consistent with previous findings (Bi et al., 2021; Li et al., 2020). Notably, some of these brain areas have been identified by functional neuroimaging studies to be crucially involved in successful EF (Niendam et al., 2012; Rodríguez-Nieto et al., 2022).

### 4.3 Relationship between brain efficiency and executive functioning in older adults

Both global (i.e., efficiency of information transfer for the entire brain) and local (i.e., efficiency of information transfer for the local subnetworks) efficiency of structural brain networks showed marked positive correlations with EF in older adults in this study. In other words, better EF was linked to higher efficiency—thus to both better network integration (as indicated by global efficiency) and to better network segregation (as indicated by local efficiency). This is consistent with the idea that successful EF relies on intact white matter connections for efficient information transfer (Bennett and Madden, 2014; Fjell et al., 2017; Madden et al., 2012).

Similar to the link between age differences and white matter measures, local efficiency was related to EF in an aggregated measure across all brain regions under investigation. In addition, more fine-grained analyses showed associations between regional efficiency and EF to be centered on fronto-parietal and subcortical (including hippocampus) regions, which is consistent with the areas implicated in EF in functional neuroimaging work (Niendam et al., 2012; Rodríguez-Nieto et al., 2022). These areas also correspond closely to the set of brain regions where better EF was reported to correlate with higher structural regional connectivity in previous work (Wen et al., 2011).

Reduced network efficiency might hinder successful executive performance. Specifically, it can be speculated that the reduced quality of structural networks (as reflected in reduced global and local network efficiency) hampers the precise recruitment of the appropriate subnetworks when performing a cognitively challenging task, which may render information processing less efficient and more erroneous. Moreover, (haphazard) excessive recruitment of additional brain areas might be facilitated, further reducing the specificity of neural recruitment and increasing inappropriate interference. In addition, the lower efficiency of local structural networks found in the present data may align with the notion that functional connectivity *within* networks is often decreased in older age (Deery et al., 2023). Note that such interpretations are speculative and require functional and structural network characteristics to be studied simultaneously.

As global, local and regional efficiency of a large set of nodes were commonly related to age and executive functioning, the next step was to examine whether networks efficiency mediated the relationship between age and executive functioning in older adults.

### 4.4 Mediation of age-associated differences in brain efficiency on age-associated differences in executive functioning in older adults

Global efficiency (i.e., efficiency of information transfer for the entire brain) did not significantly mediate age-associated EF decrease. However, our analyses revealed a significant contribution of local efficiency (i.e., efficiency of information transfer in local subnetworks averaged across all 84 brain areas under investigation) to the relationship between age and EF in older adults. This mediation of age-associated EF differences by local efficiency was driven by the regional efficiency of a number of brain regions. Specifically, we found significant mediating effects of regional efficiency for frontal (left superior frontal gyrus and medial orbitofrontal gyrus; and right pars orbitalis), parietal (bilateral precuneus), occipital (left lingual gyrus) and subcortical (bilateral hippocampus; left thalamus) areas. Some of these brain areas correspond to the fronto-parietal and subcortical areas that are often associated with EF in functional neuroimaging (Niendam et al., 2012; Rodríguez-Nieto et al., 2022).

In the current dataset, local efficiency (reflecting segregation) was identified as a mediator of the relationship between age and EF, whereas no such relationship was detected for global efficiency (reflecting integration). Given the absence of a significant mediation for global efficiency (which does not allow for firm conclusions in either direction), it remains unclear if this pattern of results reflects that local efficiency is more crucial than global efficiency in explaining the age-associated EF difference that we found here. To the extent that (a) the associations between inter-individual differences found here are reflective of intra-individual processes (but see Borsboom et al., 2009), and (b) the absence of a significant mediation of the age-EF relationship by global efficiency reflects that global efficiency truly has no mediating role in that relationship, one may speculate that local efficiency is especially important in the age-related differences in EF functioning. Supplementary analyses further showed that clustering -another measure of segregation- was also a significant mediator between age and EF. These results suggest that segregation is essential in mediating the association between aging and executive functioning. It should also be noted that the indirect mediation effects observed here were rather small, indicating that other factors are relevant in determining the association between age and EF. Longitudinal observation studies may show if alterations in structural brain networks, as they occur during aging, predict EF deterioration (see also Fjell et al., 2017; Westlye et al., 2010). If so, structural brain network metrics might serve as early indicators of age-associated EF decline.

### 4.5 Strengths and limitations

The strengths of this study are its relatively large sample size, allowing for the coverage of a broad age range within older adults and the combination of a solid EF measure (as derived from latent variables) with one of the most recent state-of-the-art techniques in the analysis of the diffusion neuroimaging data (i.e., CSD in combination with ACT and SIFT2). For the assessment of EF, we utilized a large battery of neuropsychological tasks in order to extract a latent measure of EF (Friedman et al., 2016), which helps overcoming the limitations associated with single-task measures (Miyake et al., 2000; Miyake and Friedman, 2012).

For the assessment of structural brain networks, we obtained dMRI data that we analyzed using the constrained spherical deconvolution (CSD) model in combination with anatomically-constrained tractography (ACT) and spherical-deconvolution informed filtering of tractograms (SIFT2; Smith et al., 2012, 2015a; Tournier et al., 2010). These advanced analysis techniques mitigate methodological limitations with regard to crossing fibers that constitute a limitation for more traditional approaches (e.g., fractional anisotropy measures derived from diffusion tensor imaging) and have been shown to provide more reproducible and biologically meaningful connectomes (Smith et al., 2015b). Hence, this study complements the existing literature, in that it combines a rigorous approach to EF assessment with advanced techniques for the analysis of structural brain network topology in a graph-theoretical approach (see also Madden et al., 2012).

When interpreting these results, the following limitations should be considered. First, these data are correlational in nature. Hence, they do not allow for mechanistic conclusions regarding the direct involvement of the brain areas in cognitive processes, as discussed here, and individual differences in structural network parameters should not be mistaken to reflect proximate causes of EF differences (Borsboom et al., 2009). In addition, given the cross-sectional nature of these findings, it cannot be concluded based on the current data alone that reduced network efficiency in older as compared to younger individuals (as reflected in the correlations of age and network efficiency metrics) results from the aging process *per se*. Nevertheless, longitudinal research has identified age-related decreases of structural brain networks (Alloza et al., 2018; Fjell et al., 2017), and our results are compatible with the notion that structural brain networks are subject to decline during aging. Hence, interventions targeting the preservation of structural brain networks, and more specifically local efficiency (e.g., cognitive training interventions, Caeyenberghs et al., 2016), may be an interesting route for future research. Simultaneous assessment of the functional connectome in such studies would also allow for evaluating how differences in structural and functional connectomes are temporally related. This may be informative for generating mechanistic hypotheses regarding the consequences of age-related decline in structural connectivity. In addition, functional studies would allow for the assessment of more indirect functional connectivity and information transfer (e.g., two nodes being connected via a third node), which is not possible based on the analysis of direct connections of the structural connectome reported here.

Second, the current dataset does not allow for a comparison of the established relationships with a younger control group. Hence, it remains unclear whether similar mediation exists at young age (i.e., < 60 years of age) or whether this pattern is typical for the subpopulation of older adults (i.e., ≥60 years of age). Still, our data provide valuable information regarding the link between age, structural brain connectivity, and EF for older adults and thus contribute to the understanding of EF and the role of brain networks in the aging population.

The current study did not systematically address sex differences in the relationships between age, brain efficiency parameters, and EF. In this dataset, neither EF, nor global or local efficiency differed significantly between the sexes (all *p* >0.372). Future work may further explore the nature and magnitude of sex effects, potentially in combination with neurochemical assessments. When addressing such sex effects, the role of postmenopausal shifts in neurotransmission should be considered by including a young control group and/or employing a longitudinal design. Finally, it should be noted that our choice to use Desikan's atlas for brain parcellation and grouping according to Klein and Tourville (2012) resulted in this particular architecture of the structural connectome. The automatization for the use of this atlas has shown to be anatomically valid and reliable (Desikan et al., 2006) and has been widely adopted, which allows cross-study comparability. Nonetheless, alternative parcellations and groupings, for instance according to functional networks (Yeo et al., 2011), would also have been conceivable, and may have resulted in somewhat different conclusions. Analyses focused on networks or distinct tracts that are more specifically related to EF, rather than a global approach as employed here, may provide a more detailed perspective on microstructural alterations in these regions, and increase the sensitivity to detect differences between EF subdomains.

## 5 Conclusion

This study suggests that the decreased executive functioning performance with age in older adults is mediated by changes in the local efficiency of structural connectivity. That a similar mediation effect is observed from the clustering analysis while the mediation effect from global efficiency is lacking, suggests that the decrease in network segregation in older adults is associated with higher-order cognitive functions, whereas no such relationship is being observed for network integration. Further evidence suggests that this effect is mainly driven by a lower connectivity efficiency in particular brain regions (superior frontal gyrus, orbitofrontal regions, precuneus, lingual gyrus, hippocampus and thalamus). These nodes may be critical for executive functioning and may serve as processing hubs. Future studies could possibly reveal which biological and environmental factors influence structural segregation.

## Data Availability

The datasets presented in this study can be found in OSF online repository: https://osf.io/hxr38/?view_only=b95809d059f642e48e67e6826b64d534.
