# Supplementary material for: Efficiency of structural brain networks mediates age-associated differences in executive functioning in older adults
Source: Front Aging Neurosci. 2025 Jul 8;17:1593868. doi: 10.3389/fnagi.2025.1593868 (PMC12279719; doi:10.3389/fnagi.2025.1593868)
Supplement: Supplementary file 1 [file Supplementary_file_1.docx]

**Supplementary materials**

**Table S1**

*Factor loadings for EF model.*

|  | **Estimate** | **Standard error** | ***z*** | ***p*** |
| --- | --- | --- | --- | --- |
| **Common EF** |  |  |  |  |
| Antisaccade task | .73 | .13 | 5.50 | < .001 |
| Number-Stroop task | -.01 | .13 | -0.07 | .942 |
| Stop-signal task | .27 | .13 | 2.13 | .033 |
| Category-switch task | .38 | .13 | 2.86 | .004 |
| Color-shape task | .09 | .14 | 0.63 | .526 |
| Number-letter task | .57 | .13 | 4.46 | < .001 |
| Digit-span task | .24 | .13 | 1.81 | .070 |
| Keep track task | .10 | .14 | 0.77 | .439 |
| Spatial 2-back task | .58 | .13 | 4.61 | < .001 |
| **Shifting-specific** |  |  |  |  |
| Category-switch task | .49 | .26 | 1.88 | .060 |
| Color-shape task | .30 | .20 | 1.52 | .128 |
| Number-letter task | .37 | .21 | 1.79 | .073 |
| **Updating-specific** |  |  |  |  |
| Digit-span task | .67 | .21 | 3.14 | .002 |
| Keep track task | .54 | .19 | 2.84 | .005 |
| Spatial 2-back task | .31 | .13 | 2.35 | .019 |

*Note*. Fit indices: χ^2^(21) = 21.60, *p* = .423, CFI > .99, RMSEA = .02, SRMR = .07.

**Table S2.** *Average regional efficiency and across regions for different brain lobes.*

| **Lobe** | **Average regional efficiency (SD)** |
| --- | --- |
| Frontal | 2822.29 (1746.81) |
| Left Caudal Middle Frontal | 2869.47 (470.73) |
| Right Caudal Middle Frontal | 2728.67 (458.82) |
| Left Lateral Orbitofrontal Gyrus | 2638.64 (439.99) |
| Right Lateral Orbitofrontal Gyrus | 2498.64 (417.94) |
| Left Medial Orbitofrontal Gyrus | 1589.15 (353.02) |
| Right Medial Orbitofrontal Gyrus | 1314.89 (297.25) |
| Left Paracentral Gyrus | 1929.09 (268.54) |
| Right Paracentral Gyrus | 2197.23 (351.21) |
| Left Pars Opercularis | 2412.26 (363.31) |
| Right Pars Opercularis | 2097.91 (318.56) |
| Left Pars Orbitalis | 1340.81 (242.82) |
| Right Pars Orbitalis | 1479.69 (298.02) |
| Left Pars Triangularis | 1787.64 (339.19) |
| Right Pars Triangularis | 2016.07 (349.51) |
| Left Precentral Gyrus | 5111.71 (641.31) |
| Right Precentral Gyrus | 4773.21 (629.52) |
| Left Rostral Middle Frontal Gyrus | 3680.77 (738.81) |
| Right Rostral Middle Frontal Gyrus | 3799.03 (744.52) |
| Left Superior Frontal Gyrus | 7050.63 (1009.57) |
| Right Superior Frontal Gyrus | 6915.42 (1012.71) |
| Left Frontal Pole | 893.82 (171.07) |
| Right Frontal Pole | 965.83 (171.04) |
| Parietal | 3645.49 (953.35) |
| Left Inferior Parietal Gyrus | 3219.37 (637.13) |
| Right Inferior Parietal Gyrus | 3288.94 (749.21) |
| Left Postcentral Gyrus | 3171.11 (480.36) |
| Right Postcentral Gyrus | 2907.91 (450.46) |
| Left Precuneus | 3713.71 (672.28) |
| Right Precuneus | 3994.89 (660.63) |
| Left Superior Parietal Gyrus | 5289.35 (899.91) |
| Right Superior Parietal Gyrus | 5303.01 (950.34) |
| Left Supramarginal Gyrus | 2923.09 (496.58) |
| Right Supramarginal Gyrus | 2643.54 (591.33) |
| Temporal | 1335.06 (893.06) |
| Left Banks of the Superior Temporal Sulcus | 744.63 (157.93) |
| Right Banks of the Superior Temporal Sulcus | 737.83 (132.88) |
| Left Entorhinal Cortex | 512.76 (123.77) |
| Right Entorhinal Cortex | 477.29 (108.93) |
| Left Fusiform Gyrus | 1369.49 (309.08) |
| Right Fusiform Gyrus | 1205.87 (252.45) |
| Left Inferior Temporal Gyrus | 1997.39 (402.48) |
| Right Inferior Temporal Gyrus | 1822.51 (292.46) |
| Left Middle Temporal Gyrus | 2448.73 (407.81) |
| Right Middle Temporal Gyrus | 2649.56 (421.34) |
| Left Parahippocampal Gyrus | 601.01 (116.11) |
| Right Parahippocampal Gyrus | 650.11 (113.01) |
| Left Superior Temporal Gyrus | 2972.77 (401.02) |
| Right Superior Temporal Gyrus | 2870.42 (374.21) |
| Left Temporal Pole | 1062.29 (175.37) |
| Right Temporal Pole | 982.84 (187.15) |
| Left Transverse Temporal Gyrus | 538.05 (127.07) |
| Right Transverse Temporal Gyrus | 387.45 (98.33) |
| Occipital | 1564.46 (639.11) |
| Left Cuneus | 1130.33 (235.74) |
| Right Cuneus | 1281.09 (249.79) |
| Left Lateral Occipital Gyrus | 2638.64 (439.99) |
| Right Lateral Occipital Gyrus | 2498.64 (417.93) |
| Left Lingual Gyrus | 1325.62 (212.43) |
| Right Lingual Gyrus | 1515.18 (234.12) |
| Left Pericalcarine Cortex | 1033.54 (266.57) |
| Right Pericalcarine Cortex | 1092.68 (224.09) |
| Insular & Cingulate | 1790.66 (647.39) |
| Left Caudal Anterior Cingulate | 1353.11 (315.83) |
| Right Caudal Anterior Cingulate | 1473.92 (323.82) |
| Left Isthmus Cingulate Gyrus | 2222.75 (360.84) |
| Right Isthmus Cingulate Gyrus | 2019.24 (297.32) |
| Left Posterior Cingulate Gyrus | 1760.97 (321.84) |
| Right Posterior Cingulate Gyrus | 1741.21 (283.59) |
| Left Rostral Anterior Cingulate Gyrus | 1037.32 (246.44 |
| Right Rostral Anterior Cingulate Gyrus | 846.26 (154.54) |
| Left Insula | 2846.46 (456.04) |
| Right Insula | 2605.42 (467.87) |
| Subcortical | 2319.96 (1875.38) |
| Left Thalamus | 5704.83 (604.75) |
| Right Thalamus | 5629.21 (596.98) |
| Left Caudate | 2268.12 (457.33) |
| Right Caudate | 2223.02 (530.18) |
| Left Putamen | 4249.49 (547.64) |
| Right Putamen | 3966.39 (492.77) |
| Left Pallidum | 2126.81 (378.49) |
| Right Pallidum | 2283.59 (365.21) |
| Left Hippocampus | 995.13 (294.59) |
| Right Hippocampus | 1153.92 (319.68) |
| Left Amygdala | 384.17 (173.18) |
| Right Amygdala | 425.89 (237.69) |
| Left Accumbens | 575.22 (156.81) |
| Right Accumbens | 493.73 (132.05) |
| Cerebellum | 2976.86 (143.61) |
| Left Cerebellum | 2875.31 (334.96) |
| Right Cerebellum | 3078.41 (343.81) |

*Note.* Standard deviation (SD) given in parentheses. *N* = 84.

**Supplementary Analyses with Other Global Metrics**

The descriptive statistics of density, clustering, modularity, and strength are presented in Table S3, and the correlation matrices showing the associations between age, Common EF, and these metrics are shown in Table S4. In sum, all global metrics, with the exception of modularity, showed a negative and significant correlation with age and positive and significant correlation with Common EF. The mediation analyses showed that clustering significantly mediated the relationship between age and Common EF (Table S5).

The descriptive statistics for the strength of each node is presented in Table S6. The correlation of the strength of these nodes with age and with Common EF is presented in Tables S7 and S8, respectively. Given the high correlation between efficiency and strength of the nodes (average *r* = .82, *p* < .01), the results are highly similar to those observed in the analyses with regional efficiency. Still, it is noteworthy that the efficiency of a considerable number of nodes showed a correlation with age and with Common EF, but their strength did not have a significant correlation with the same variables (nine nodes for the correlation with age, and 14 nodes for the correlation with Common EF). Mediation analyses showed that only the strength of four nodes (left lateral orbitofrontal gyrus, left superior frontal gyrus, left precuneus, and right pars orbitalis) significantly mediated the relationship between age and Common EF (Table S9). This contrasts with the efficiency of nine nodes that mediated the relationship between age and Common EF (Table 3).

Table S3. Descriptive statistics of main global network metrics

| Network metric  *Description* | Mean  (sd) |
| --- | --- |
| Density  *(Fraction of present connections to possible connections)* | .97  (.02) |
| Clustering  *(Fraction of node’s neighbors that are neighbors of each other)* | 1779.21  (191.11) |
| Modularity  *(The degree to which the network may be subdivided into delineated groups; Girvan-Newman algorithm)* | .42  (.02) |
| Strength  *(The sum of weights of links connected to the node)* | 39146525.21 (4247616.94) |
| Global efficiency  *(The average inverse shortest path length in the network)* | 20287.68  (2641.57) |
| Local efficiency (average)  *(The global efficiency computed on the neighborhood of a node)* | 2254.09  (240.16) |

Table S4. Correlation among Age, Common EF and network metrics (controlling for Sex and Education).

|  | Age | EF | Density | Clustering | Modularity | Strength | E_glob_ | E_loc_ |
| --- | --- | --- | --- | --- | --- | --- | --- | --- |
| Age | 1 | -.52  <.001 | -.27  .01 | -.39  <.001 | -.01  .94 | -.41  <.001 | -.42  <.001 | -.42  <.001 |
| EF |  | 1 | .29  .008 | .36  .001 | .04  .71 | .34  .002 | .33  .003 | .37  <.001 |
| Density |  |  | 1 | .44  <.001 | -.49  <.001 | .16  .15 | .061  .59 | .36  <.001 |
| Clustering |  |  |  | 1 | -.18  .11 | .88  <.001 | .73  <.001 | .98  <.001 |
| Modularity |  |  |  |  | 1 | .12  .27 | .21  .06 | -.12  .31 |
| Strength |  |  |  |  |  | 1 | .94  <.001 | .94  <.001 |
| E_glob_ |  |  |  |  |  |  | 1 | .82  <.001 |
| E_loc_ |  |  |  |  |  |  |  | 1 |

E_glob_ – Global efficiency; E_loc_ – average local efficiency

Table S5. Mediation analyses for the relationship between Age and Executive Functions with Network metrics as mediators.

| **Region** | **β_c_** | **β_c’_** | **β_ab_** | **Boot SE** | **Boot LLCI** | **Boot ULCI** | **β_a_** | **β_b_** |
| --- | --- | --- | --- | --- | --- | --- | --- | --- |
| ***Left Hemisphere*** |  |  |  |  |  |  |  |  |
| Density  Clustering | -.53***  -.53*** | -.49***  -.46*** | -.05  -.07 | .03  .04 | -.13  -.16 | .003  -.005 | -.26*  -.41*** | .17ᶧ  .17ᶧ |
| Modularity | -.53*** | -.53*** | -.0003 | .01 | -.03 | .21 | -.01ᶧ | .04ᶧ |
| Strength  Global Efficiency | -.53***  -.53*** | -.47***  -.47*** | -.07  -.06 | .05  .05 | -.17  -.17 | .01  .02 | -.42***  -.44*** | .16ᶧ  .13ᶧ |

*Note.* Sex and education were introduced as covariates. β = standardized regression coefficient; CI = bias-corrected 95% confidence interval; Boot SE, LLCI, and ULCI = mediation effect’s standard error, lower, and upper limit of CI obtained by bootstrapping (n = 5000).

*** *p* < .001; ** *p* < .005; * *p* < .050; ^Ϯ^ *p* < .100

**Table S6.** *Average regional strength and across regions for different brain lobes.*

| **Lobe** | **Average regional strength (SD)** |
| --- | --- |
| Frontal | 594437.61 (545767.87) |
| Left Caudal Middle Frontal | 654367.42 (112222.35) |
| Right Caudal Middle Frontal | 613424.63 (115282.06) |
| Left Lateral Orbitofrontal Gyrus | 236041.93 (60555.49) |
| Right Lateral Orbitofrontal Gyrus | 239448.93 (62108.53) |
| Left Medial Orbitofrontal Gyrus | 202199.26 (52032.32) |
| Right Medial Orbitofrontal Gyrus | 182005.97 (45374.37) |
| Left Paracentral Gyrus | 372297.59 (71341.11) |
| Right Paracentral Gyrus | 435047.41 (89216.29) |
| Left Pars Opercularis | 457370.81 (87399.95) |
| Right Pars Opercularis | 383802.13 (74584.25) |
| Left Pars Orbitalis | 197148.19 (38443.35) |
| Right Pars Orbitalis | 221144.45 (52751.99) |
| Left Pars Triangularis | 262550.51 (61655.39) |
| Right Pars Triangularis | 313515.94 (66907.17) |
| Left Precentral Gyrus | 1525347.84 (205146.11) |
| Right Precentral Gyrus | 1405815.25 (198097.89) |
| Left Rostral Middle Frontal Gyrus | 769352.54 (157152.71) |
| Right Rostral Middle Frontal Gyrus | 770289.21 (163725.66) |
| Left Superior Frontal Gyrus | 1864510.55 (278992.23) |
| Right Superior Frontal Gyrus | 1764003.75 (261393.89) |
| Left Frontal Pole | 98637.98(24697.66) |
| Right Frontal Pole | 109304.88 (22522.04) |
| Parietal | 839523.77 (229465.45) |
| Left Inferior Parietal Gyrus | 859565.41 (155120.04) |
| Right Inferior Parietal Gyrus | 887964.51 (155198.24) |
| Left Postcentral Gyrus | 858982.65 (136001.31) |
| Right Postcentral Gyrus | 761643.51 (114727.16) |
| Left Precuneus | 580077.08 (108579.52) |
| Right Precuneus | 639667.69 (104616.05) |
| Left Superior Parietal Gyrus | 1204063.34 (193658.11) |
| Right Superior Parietal Gyrus | 1244371.77 (196477.81) |
| Left Supramarginal Gyrus | 748024.45 (121398.19) |
| Right Supramarginal Gyrus | 610877.25 (122034.15) |
| Temporal | 230906.81 (170019.92) |
| Left Banks of the Superior Temporal Sulcus | 130074.73 (34141.33) |
| Right Banks of the Superior Temporal Sulcus | 101799.78 (22921.41) |
| Left Entorhinal Cortex | 71309.21 (25111.21) |
| Right Entorhinal Cortex | 63009.26 (22578.98) |
| Left Fusiform Gyrus | 302332.78 (65903.83) |
| Right Fusiform Gyrus | 290503.21 (65991.07) |
| Left Inferior Temporal Gyrus | 343616.69 (76720.73) |
| Right Inferior Temporal Gyrus | 345604.49 (59190.39) |
| Left Middle Temporal Gyrus | 479334.01 (84551.59) |
| Right Middle Temporal Gyrus | 498598.47 (84336.21) |
| Left Parahippocampal Gyrus | 83802.52 (22703.99) |
| Right Parahippocampal Gyrus | 85736.56 (15691.14) |
| Left Superior Temporal Gyrus | 506200.99 (72013.21) |
| Right Superior Temporal Gyrus | 447862.64 (68611.79) |
| Left Temporal Pole | 159239.24 (31489.28) |
| Right Temporal Pole | 145352.69 (30505.46) |
| Left Transverse Temporal Gyrus | 62188.96 (18457.21) |
| Right Transverse Temporal Gyrus | 39756.56 (10190.42) |
| Occipital | 374822.84 (170371.42) |
| Left Cuneus | 257903.97 (66391.75) |
| Right Cuneus | 334904.33 (84195.13) |
| Left Lateral Occipital Gyrus | 621812.07 (107297.96) |
| Right Lateral Occipital Gyrus | 663749.09 (112376.91) |
| Left Lingual Gyrus | 254737.94 (59569.09) |
| Right Lingual Gyrus | 341354.87 (71540.92) |
| Left Pericalcarine Cortex | 226932.66 (82966.43) |
| Right Pericalcarine Cortex | 297187.68 (89447.38) |
| Insular & Cingulate | 276621.42 (108322.19) |
| Left Caudal Anterior Cingulate | 185781.64 (51475.23) |
| Right Caudal Anterior Cingulate | 210380.61 (58512.41) |
| Left Isthmus Cingulate Gyrus | 326209.21 (67800.82) |
| Right Isthmus Cingulate Gyrus | 303840.01 (57378.29) |
| Left Posterior Cingulate Gyrus | 290493.76 (64318.12) |
| Right Posterior Cingulate Gyrus | 292205.74 (61577.74) |
| Left Rostral Anterior Cingulate Gyrus | 158018.19 (44546.41) |
| Right Rostral Anterior Cingulate Gyrus | 125410.64 (26884.46) |
| Left Insula | 459236.92 (87159.19) |
| Right Insula | 414637.42 (85547.58) |
| Subcortical | 393438.01 (379377.23) |
| Left Thalamus | 1153839.74 (146303.34) |
| Right Thalamus | 1114157.69 (136320.72) |
| Left Caudate | 410669.05 (121330.81) |
| Right Caudate | 393611.39 (134535.93) |
| Left Putamen | 703295.33 (102339.17) |
| Right Putamen | 620332.69 (94205.25) |
| Left Pallidum | 313303.68 (79960.59) |
| Right Pallidum | 342110.89 (70867.82) |
| Left Hippocampus | 136277.87 (48755.19) |
| Right Hippocampus | 144525.86 (44959.22) |
| Left Amygdala | 41360.28 (25558.81) |
| Right Amygdala | 42680.57 (33060.82) |
| Left Accumbens | 49949.49 (17636.78) |
| Right Accumbens | 42017.62 (13972.91) |
| Cerebellum | 1137204.32 (27368.35) |
| Left Cerebellum | 1117851.97 (128401.21) |
| Right Cerebellum | 1156556.66 (132005.72) |

*Note.* Standard deviation (SD) given in parentheses. *N* = 84.

Table S7. *Regions with significant Age -Strength association (controlled for sex and years of education) are listed according to hemisphere, lobe, and ascending order of p-value (FDR critical p = .026).*

| **Region** | **Lobe/Area** | ***R*** | ***p*** |
| --- | --- | --- | --- |
| ***Left Hemisphere*** |  |  |  |
| Pars Orbitalis*  Pars Triangularis*  Rostral Middle Frontal Gyrus*  Pars Opercularis*  Lateral Orbitofrontal Gyrus*  Superior Frontal Gyrus*  Precentral Gyrus* | Frontal  Frontal  Frontal  Frontal  Frontal  Frontal  Frontal | -.49  -.37  -.43  -.35  -.33  -.29  -.26 | < .001  <.001  <.001  .001  .002  .009  .017 |
| Precuneus*  Superior Parietal Gyrus*  Supramarginal Gyrus*  Inferior Parietal Gyrus* | Parietal  Parietal  Parietal  Parietal | -.38  -.36  -.29  -.35 | <.001  < .001  .001  .009 |
| Middle Temporal Gyrus*  Superior Temporal Gyrus*  Banks of the Superior Temporal Sulcus Inferior Temporal Gyrus* | Temporal  Temporal  Temporal  Temporal | -.46  -.36  -.32  -.26 | <.001  .001  .003  .016 |
| Lateral Occipital Gyrus* | Occipital | -.33 | .002 |
| Hippocampus*  Thalamus* | Subcortical  Subcortical | -.43  -.31 | <.001  .006 |
| ***Right Hemisphere*** |  |  |  |
| Rostral Middle Frontal Gyrus* | Frontal | -.49 | < .001 |
| Pars Triangularis* | Frontal | -.36 | < .001 |
| Pars Orbitalis* | Frontal | -.41 | < .001 |
| Superior Frontal Gyrus* | Frontal | -.35 | .001 |
| Precentral Gyrus* | Frontal | -.26 | .019 |
|  |  |  |  |
| Superior Parietal Gyrus* | Parietal | -.36 | < .001 |
| Precuneus* | Parietal | -.44 | < .001 |
| Postcentral Gyrus* | Parietal | -.32 | < .004 |
| Inferior Parietal Gyrus* | Parietal | -.34 | .002 |
| Entorhinal Cortex* | Temporal | -.32 | .004 |
| Superior Temporal Gyrus* | Temporal | -.37 | <.001 |
| Inferior Temporal Gyrus* | Temporal | -.35 | .001 |
| Fusiform Gyrus*  Middle Temporal Gyrus | Temporal  Temporal | -.29  -.27 | .008  .014 |
| Lateral Occipital Gyrus* | Occipital | -.47 | <.001 |
| Cuneus* | Occipital | -.27 | .017 |
| Hippocampus* | Subcortical | -.51 | < .001 |
| Accumbens* | Subcortical | -.29 | .009 |

(*) Regions that also had a correlation with Global efficiency (after FDR)

**Table S8.** *Regions with significant Common EF-Strength association (controlled for sex and years of education) are listed according to hemisphere, lobe, and ascending order of p-value (FDR critical p = .027).*

| **Region** | **Lobe/Area** | ***r*** | ***p*** |
| --- | --- | --- | --- |
| ***Left Hemisphere*** |  |  |  |
| Superior Frontal Gyrus* | Frontal | .37 | <.001 |
| Medial Orbitofrontal Gyrus* | Frontal | .38 | <.001 |
| Lateral Orbitofrontal Gyrus* | Frontal | .38 | <.001 |
| Pars Orbitalis* | Frontal | .31 | .005 |
| Rostral Middle Frontal Gyrus* | Frontal | .29 | .007 |
| Precentral Gyrus*  Pars Opercularis*  Frontal Pole* | Frontal  Frontal  Frontal | .27  .25  .25 | .015  .021  .024 |
| Precuneus* | Parietal | .39 | < .001 |
| Superior Parietal Gyrus* | Parietal | .28 | .011 |
| Inferior Parietal Gyrus* | Parietal | .28 | .012 |
| Supramarginal Gyrus* | Parietal | .26 | .019 |
| Banks of the Superior Temporal Sulcus*  Transverse Temporal Gyrus*  Middle Temporal Gyrus* | Temporal  Temporal  Temporal | .29  .28  .27 | .008  .011  .012 |
| Lingual Gyrus* | Occipital | .34 | .002 |
| Hippocampus* | Subcortical | .34 | .002 |
| Thalamus* | Subcortical | .32 | .003 |
| Caudate*  Putamen* | Subcortical  Subcortical | .29  .25 | .008  .022 |
| ***Right Hemisphere*** |  |  |  |
| Pars Orbitalis*  Lateral Orbitofrontal Gyrus*  Precentral Gyrus* | Frontal  Frontal  Frontal | .37  .28  .27 | <.001  .012  .013 |
| Superior Frontal Gyrus* | Frontal | .26 | .016 |
| Pars Triangularis* | Frontal | .26 | .018 |
| Rostral Middle Frontal Gyrus* | Frontal | .26 | .019 |
| Precuneus* | Parietal | .39 | < .001 |
| Middle Temporal Gyrus*  Inferior Temporal Gyrus* | Temporal  Temporal | .28  .27 | .011  .012 |
| Lateral Occipital Gyrus* | Occipital | .27 | .017 |
| Hippocampus* | Subcortical | .41 | < .001 |
| Thalamus* | Subcortical | .31 | .004 |

*Note. r*-values are rounded to two decimals. Assignment of lobes/areas according to Desikan et al. (2006)

(*) Regions that also had a correlation with Global efficiency (after FDR)

**Table S9.** *Brain regions with Strength significantly mediating the age-associated decline in Common EF in older adults. The regions are listed according to hemisphere, and lobe. Zero outside the CI indicates significance of the mediation effect (*β*_ab_).*

| **Region** | | **Lobe/Area** | **β_c_** | **β_c’_** | **β_ab_** | **Boot SE** | **Boot LLCI** | **Boot ULCI** | **β_a_** | **β_b_** |
| --- | --- | --- | --- | --- | --- | --- | --- | --- | --- | --- |
| ***Left Hemisphere*** | |  |  |  |  |  |  |  |  |  |
| Lateral Orbitofrontal Gyrus  Superior Frontal Gyrus* | | Frontal  Frontal | -.53***  -.53*** | -.46*  -.46* | -.07  -.07 | .04  .04 | -.15  -.15 | -.01  -.007 | -.29**  -.29** | .22*  .24* |
|  |  |  |  |  |  |  |  |  |  |  |
| Precuneus* | | Parietal | -.53*** | -.44*** | -.09 | .04 | -.18 | -.02 | -39*** | .23* |
| ***Right Hemisphere*** | |  |  |  |  |  |  |  |  |  |
| Pars Orbitalis* | | Frontal | -.53*** | -.45*** | -.08 | .04 | -.16 | -.008 | .42*** | .19^Ϯ^ |

*Note.* β = standardized regression coefficient; CI = bias-corrected 95% confidence interval; Boot SE, LLCI, and ULCI = mediation effect’s standard error, lower, and upper limit of CI obtained by bootstrapping (n = 5000). Assignment of lobes/areas according to Desikan et al. (2006).

*** *p* < .001; ** *p* < .005; * *p* < .050; ^Ϯ^ *p* < .100

Table S10. Correlation between the Shifting and Updating factors and Network Efficiency metrics.

|  | Age | Shifting | Updating | Global efficiency | Local  efficiency |
| --- | --- | --- | --- | --- | --- |
| Age | 1 | -.26* | .02 | -.38** | -.41** |
| Shifting |  | 1 | -.03 | .01 | .08 |
| Updating |  |  | 1 | -.02 | .02 |
| Global efficiency |  |  |  | 1 | .82** |
| Local efficiency |  |  |  |  | 1 |

** *p* < .001; * *p* < .05
